# A Decade of Experience in Diagnostic and Conservative Treatment of Endometrial Malignancy—Oncologic and Obstetrical Outcomes from a Referral Oncofertility Center

**DOI:** 10.3390/diagnostics15111388

**Published:** 2025-05-30

**Authors:** Katarina Stefanovic, Jelena Dotlic, Igor Pilic, Branislav Milosevic, Olga Mihaljevic, Aleksandra Beleslin, Aleksandar Stefanović

**Affiliations:** 1 Clinic for Obstetrics and Gynecology, University Clinical Centre of Serbia, Dr Koste Todorovica 26, 11000 Belgrade, Serbia; drenadot@gmail.com (J.D.); gak.stefanovic@gmail.com (A.S.); 2 Medical Faculty, University of Belgrade, Dr Subotica 8, 11000 Belgrade, Serbia

**Keywords:** endometrial cancer, conservative treatment, oncologic outcome, obstetrical outcome

## Abstract

**Background/Objectives:** This study aimed to investigate oncologic and obstetrical outcomes of patients conservatively treated for atypical hyperplasia (AH), endometrial intraepithelial neoplasm (EIN), and early endometrial cancer (EC), as well as factors that influence these outcomes. **Methods**: This study included 87 women conservatively treated due to AH/EIN and well-differentiated endometrioid EC confined only to the endometrium during past 10 years. Therapy type, course, and duration were registered. The response totherapy after 12 months (remission vs. disease persisting or progressing) was considered as the oncologic outcome. All attempted and achieved pregnancies, along with conception method, gestational week, and delivery type, were recorded. The obstetrical outcomes were classified as adverse (miscarriage) or successful (healthy child). **Results**: All patients received LNG-IUD along with GnRHa and, if indicated, metformin. Complete remission was achieved in 74.7% of patients. The disease was persisting in 17.2% and progressing in 3.5% of patients, while recurrence was registered in 4.6% of patients. Radical surgery during follow-up was indicated in 15% of patients due to condition deterioration. Pregnancy was attempted by 29.9% of patients, out of which nine succeeded (34.6%). There were two early miscarriages, while the remaining seven pregnancies ended in a term delivery of a healthy child, mostly by planned cesarean section. The only predictor of long-term disease remission was malignancy-free control histological findings. Better therapy response and achieving remission in shorter time were predictors of good obstetrical outcome. **Conclusions**: This study proved the efficacy and safety of current protocols for AH/EIN/EC conservative treatment and indicated that adequate early (6-month) response totherapy has the most importance for long-term remission and pregnancy achievement.

## 1. Introduction

Endometrial cancer (EC) is the fourth most commonly diagnosed cancer and the sixth most common cause of death due to cancer in women. It is the most common gynecological malignancy in developed countries, with the highest incidence in North America (21.1 per 100,000 women). The incidence of EC in Eastern Europe is 20.2 per 100,000 women [1,2].

Although EC is mostly diagnosed in postmenopausal women (90% in women aged ≥50 years), up to 25% are found in premenopausal women. Around 5% of endometrial cancers occur in women younger than 45 years, with 2% found in women aged 20–25. In Serbia, 3.4% of women with endometrial cancer are younger than 45. The majority of younger women with EC (almost 70%) are nulliparous or still planning to reproduce [1,2,3,4,5]. Currently the combination of transvaginal grey scale ultrasound and color Doppler examinations is generally accepted as the first line method for endometrial assessment because of their noninvasive nature but highly reliability and availability. The endometrial thickening, low resistance, and pulsatility indices, as well as peak systolic velocity of the uterine artery, are associated with malignancy. Definite diagnosis is achieved by histological analysis of endometrial samples obtained by hysteroscopy or curettage [6,7].

Atypical endometrial hyperplasia (AH) is a precancerous condition, while endometrial intraepithelial neoplasm (EIN) is the premalignant endometrial glandular lesion. If not treated, 25–50% of complex endometrial hyperplasia cases with atypia will evolve into EC. AH and EIN have similar clinical presentations as EC [1,2,3,4,5].

For AH, EIN, and EC cases in older women, the therapy of choice is surgery (total hysterectomy, bilateral salpingo-oophorectomy, and lymph node assessment/dissection) [1,3,8,9]. However, for younger women who have not yet finished reproduction, treatment must be reconsidered. More than 80% of younger women present with either a precancerous condition or an initial stage of malignancy that has good survival and favorable prognostic features [8,9,10].

International guidelines suggest conservative fertility-sparing procedures, but only after careful counseling and with close follow-up. Various treatment strategies based on different hormonal preparations are currently used for younger women [1,6,7]. Nevertheless, conservative management poses a risk for cancer persistence, progression, recurrence, and even adverse outcomes [8,9,10].

This study aimed to investigate the oncologic and obstetrical outcomes of patients treated for AH, EIN, and early EC in the Clinic for Obstetrics and Gynecology University Clinical Centre of Serbia, as well as the factors that influence these outcomes.

## 2. Materials and Methods

This study was undertaken over a period of 10 years (1 January2014–1 January 2023) at the Clinic for Obstetrics and Gynecology University Clinical Centre of Serbia Medical Faculty, University of Belgrade. It was approved by the Ethical Committee of the University Clinical Centre of Serbia (30 November 2023; No. 694/11). Inclusion criteria were histologically confirmed AH, EIN, and/or well-differentiated endometrioid EC confined only to the endometrium with no myometrial invasion or extrauterine disease on imaging, strong desire to preserve fertility, age ≤45 years, no contraindication to medical treatment, and signed informed consent.

The condition was histologically confirmed by two experienced pathologists for every patient after curettage or hysteroscopy. EC is currently divided into four molecular types according to genomic instability: (1) POLE/ultramutated, (2) microsatellite unstable and mismatch repair deficient/hypermutated, (3) copy-number low, and (4) copy-number high, largely corresponding to TP53-mutant serous and high-grade endometrioid carcinomas. Cytologic atypia superimposed on endometrial hyperplasia defines AH/EIN. The WHO 2020 classification assesses AH and EIN together, specifying as essential diagnostic criteria crowded glandular architecture with altered epithelial cytology distinct from non-neoplastic glands. Additional criteria include sufficient size that artifact can be excluded, loss of immunoreactivity for PTEN, PAX2, or mismatch repair proteins. Therefore, to confirm the diagnosis, during histological analyses endometrial specimens of our patients were tested for PAX 2 and PTEN antigen status, while progesterone receptor status was determined in special cases [11].

EC was staged according to the International Federation of Gynecology and Obstetrics (FIGO) system. Indications and the type of initial diagnostic procedure (hysteroscopy or curettage) were noted. A baseline pretreatment assessment for all patients included body mass index (BMI) calculation, family history, personal medical and gynecological history, and a thorough gynecological examination, including colposcopy and Papanicolaou testing, laboratory testing (blood count, coagulation factors, biochemical analyses such as glucose level, liver function tests and electrolytes, tumor markers cancer antigen 125 (Ca 125) and human epididymis protein 4 (HE4) with Risk of Ovarian Malignancy Algorithm (ROMA) calculation, basal hormonal status and anti-Mullerian hormone, glucose tolerance testing), and imaging of the pelvis and abdomen (ultrasound—US—and magnetic resonance imaging—MRI—for myometrial invasion assessment), breast ultrasound, chest X-ray, and genetic testing.

Patients had detailed consultations regarding the fact that fertility-sparing therapy is unconventional treatment that carries numerous risks but also potentially enables reproduction. All patients provided signed informed consent for all therapeutic procedures, obstetrical management, and this study.

We registered the therapy type, course, and duration. In our oncofertility center for patients with AH/EIN, and EC in the first stage, conservative fertility-sparing treatment included hormonal therapy (levonorgestrel-releasing intrauterine device—LNG-IUD—and gonadotropin-releasing hormone analogs—GnRHa) for 6–9 months. If indicated and accessible during hysteroscopy, a tumor resection was performed. For patients with BMI ≥25, weight reduction (mild physical activity and Mediterranean diet) was recommended. Metformin was also administered in cases of diagnosed insulin resistance and/or polycystic ovarian syndrome (PCOS).

Patients were followed up according to current standard protocols (every 3 months with laboratory tests, transvaginal US with endometrial measuring; every 6 months LNG-IUD extraction and uterine cavity visualization, and evaluation via hysteroscopy and/or curettage for endometrial sampling). More frequent endometrial sampling was indicated if the patient had symptoms (bleeding) or abnormal examination findings (especially endometrial hyperplasia on US i.e., endometrial thickness ≥8 mm). Treatment was stopped when two successive evaluations showed they were malignancy free.

For the purpose of this study, although the follow-up of patients is still ongoing, the cut-off for therapy assessment was made 12 months after therapy commencement. Therapy response was defined as complete remission (no signs of malignancy on histological examination of endometrial samples at the end of follow-up), disease persisting (no regression of the findings within six months of treatment commencement), disease progressing (stage or grade increased during follow-up), and recurrent disease (malignant cells histologically diagnosed in control endometrial samples after a period of complete remission). In this study, the response to therapy after 12 months—good (complete remission i.e., malignancy-free) vs. bad (disease persisting or progressing)—was considered the oncologic outcome of the examined patients. Nevertheless, as we had the patient data of the whole follow-up (which for some patients lasted 10 years), we opted to present them as well, but only descriptively.

LNG-IUD was used as maintenance therapy until pregnancy was attempted. Surgical radicalization was indicated in cases of disease persisting longer than 12 months, disease progression, and recurrent disease, but also for complications and/or general patient condition deterioration, as well as after childbearing or when the patient finished reproduction.

Finally, we assessed the obstetric outcomes of our patients. We recorded all attempted and achieved pregnancies. The obstetrical outcomes were classified as adverse (miscarriage) or successful (delivery of a healthy child). We also noted the method of conception (spontaneous or using assisted reproduction—ART), gestational week, and type of delivery (vaginal or cesarean section—CS).

All data were statistically analyzed using the Statistical Package for the Social Sciences (SPSS 20 IBM, Armonk, USA, 2011) software and standard methods for descriptive and analytical statistics. Chi square test (χ^2^) was used to assess differences in patient, tumor, and therapy characteristics with regard to oncologic and obstetrical outcomes. Spearman’s correlation was performed to test the associations between patient, tumor, and therapy characteristics, and oncologic and obstetrical outcomes. Binary logistic regression was applied to investigate which of the registered parameters could be the risk factors the oncologic and obstetrical outcomes. Parameters were divided into two logical groups (general and medical history patient history data, and data regarding malignancy and its therapy) and included as such in the models according to their correlations with assessed outcomes. In this way, the number of potential predictors per model was adequate for the number of patients. No co-linearity was found between the investigated parameters.

## 3. Results

**Figure 1:**
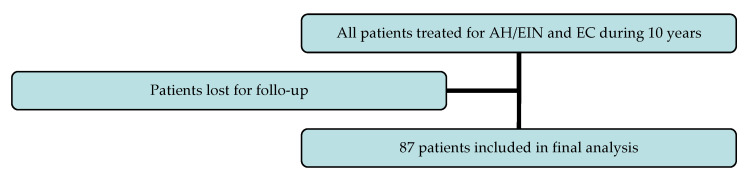
Inclusion and exclusion of patients.

During the past decade, around 120 women were conservatively treated due to AH/EIN and EC in our referral center, but some were lost duringfollow-up (Figure 1). Consequently, this study included 87 women who were 35.05 ± 25.09 years (21 to 45 years) on average. General and medical history data of patients are presented on Table 1. Their average BMI was 25.09 ± 4.68, but 44.8% were overweight and obese. The majority of women were healthy, while the most common comorbidity was hypothyreosis (14.5%). Other gynecological illnesses (almost all benignant) were registered in 14.9% of patients. Only one patient had an ovarian borderline tumor (stage IC1) that was surgically removed three years before EC diagnosis. Moreover, only a few patients had a positive family history of malignancy, and just one patient had hereditary genetic aberrations (Lynch syndrome).

In our sample, there were no women with early or late puberty (age of menarche 9 to 19 years). Menstrual cycle was regular in nearly 80% of patients. Almost all of the investigated women were nulliparous and a half of women already treated infertility. They had up to four procedures of assisted reproduction prior to the EC diagnosis.

Table 2 shows data regarding malignancy and its therapy. The most common histological finding was endometrial carcinoma G1. Diagnosis was mostly made during hysteroscopy (60.9%) performed due to endometrial polyp visualized by US. Irregular bleeding was the usual (19.5%) indication for exploratory curettage during which the malignancy was diagnosed.

Application of LNG-IUD was the treatment of choice for all investigated patients along with GnRHa, while 13.8% of patients received additional metformin. Hysteroscopic resection of endometrial lesions to the underlying myometrium was performed in two patients. The therapy was well tolerated with almost no adverse effects (three patients reported having hot flushes and nine women had spotting during first three months of therapy) and, consequently, compliance was total. During follow-up, just one patient developed hypertension. Moreover, the majority (79.5%) of women with BMI ≥25 complied with the suggested lifestyle changes, and they reduced their weight by 11.28 ± 5.4 kg (range 5 to 28 kg) on average.

All patients were regularly checked-up throughout the period of receiving therapy with gynecological and imaging examinations for 9.7 ± 3.1 months onaverage (6 to 15 months). Patients had up to four control hysteroscopies over the course of the follow-up. Endometrial sampling was performed in 55.2% of patients mostly due to suspicious US findings. Women who attempted pregnancy after complete remission continued regular controls in our institution. Moreover, follow-up of patients was ongoing up to the final radicalization of the treatment (after finishing reproduction or aborting further attempts to achieve pregnancy).

Response to therapy was good, with complete remission after 12 months in almost 75% of patients and no adverse outcomes (survival rate 100%). Around 30% of patients with complete remission stopped receiving therapy mostly one year after the initial diagnosis. Nevertheless, during the follow-up, radical surgical treatment had to be performed in 15% of patients due to the deterioration of their condition.

At the 12-month checkup, recurrent disease was registered in four (4.6%) patients and progression from EIN to EC in three (3.4%) patients. Table 3 shows characteristics of patients with disease persisting or progression. Myometrial invasion was diagnosed upon hysterectomy in two patients with recurrent disease and in one patient with disease progression. Furthermore, one of the patients with recurrent disease and myometrial invasion (two years after the initial diagnosis and one after remission) had newly discovered and removed rectal neuroendocrine tumor in the polyp 16 months after malignancy diagnosis.

Moreover, during the follow-up, two patients were diagnosed with concomitant ovarian malignancy (endometrioid adenocarcinoma of the ovary). In one patient, ovarian malignancy was diagnosed three months after therapy commencement during emergency surgery performed due to adnexal torquation. Radicalization was indicated and performed, as ovarian malignancy had already presented with local metastases (pelvic peritoneum, urinary bladder wall). In the other patient, clinical and imaging findings on the six-month control US suggested benign ovarian cysts. Therefore, laparoscopy with ovarian tumor excision was performed. Histological examination showed ovarian malignancy, which led to hysterectomy. In addition, one patient had developed cervical adenocarcinoma (10th month of follow-up), due to which initially conisation and then radical surgery were performed.

The patients from our sample attempted to achieve pregnancy in 29.9% of cases, out of which nine succeeded (34.6%) (Table 4). Women with successful pregnancies had 32 to 40 years. Although the majority of patients used ART for that purpose, pregnancy was conceived naturally in 55.6% of cases. In all cases of ART, in-vitro fertilization with embryo transfer (IVF/ET) was performed after ovarian stimulation. There were two early miscarriages, while the remaining seven pregnancies ended in a term delivery of a healthy child. All pregnancies were singleton. These pregnancies were regularly (monthly) checked according to protocol for high-risk pregnancy. Patients had no major pregnancy complications. Two patients had slight bleeding in the first trimester and one had occasional contractions, while the most severe issue was placenta previa in one patient, which therefore was delivered by CS. Delivery was mostly by planned CS in the 38.4 ± 2.7 week of gestation (37 to 40 gestational weeks) due to obstetrical indications. All children were in good condition upon birth (average Apgar score 8.3 ± 1.1) and none of them had any complications or birth defects. Characteristics of women with healthy pregnancy after conservative fertility-sparing treatment for AH/EIN and EC are shown on Table 5.

Among women who achieved pregnancy, there were no progressive or recurrent diseases. Still, after attempting pregnancy, fourteenwomen had radicalization (twelvedue to condition deterioration and two due to finishing reproduction). In one of the patients that had disease, deterioration during attempting pregnancy on histological examination after hysterectomy myometrial invasion was diagnosed.

In our study, complete disease remission (positive oncologic outcome) correlated with absence of myometrial invasion initially and throughout follow-up (*p* = 0.039) and malignancy-free all control endometrial histological findings (*p* = 0.041). Successful pregnancy and term delivery (positive obstetrical outcome) correlated with not having infertility issues prior to malignancy (*p* = 0.007) and faster achievement of complete disease remission (*p* = 0.001).

Regression analysis showed that the only predictor of long-term disease remission in conservatively treated patients with AH/EIN and EC was malignancy-free histological finding on first control endometrial sampling (6 months after therapy commencement) (Table 6). Accomplishing complete remission in a shorter time was the predictor of achieving pregnancy and delivering a healthy child (Table 7).

## 4. Discussion

In this study were assessed outcomes of patients treated during the past decade with the alternative investigational conservative protocol that has just lately been accepted as standard for AH/EIN and EC fertility-sparing treatment. Moreover, we determined which patient, tumor, and treatment characteristics have the most impact on oncologic and obstetrical outcomes. The study findings showed that both oncologic and obstetrical outcomes of patients treated according to currently accepted protocol for AH, EIN, and early EC fertility-spearing therapy can be quite good. Absence of myometrial invasion and good control histological finding are the most important for prolonged disease remission. Pregnancy achievement and term delivery correlate with not having infertility issues prior to malignancy, better response totherapy, and achieving remission in a shorter time.

Conservative fertility-sparing treatment for AH, EIN, and early-stage EC in young women who have not yet finished reproduction has widely been accepted as effective oncologic treatment which can enable further childbearing [12,13]. According to current protocols, candidates for fertility-sparing management are women younger than 40 years with premalignant disease or malignancy confined to the endometrium, well-differentiated endometrioid cancer, who are highly motivated to maintain their reproductive function. The assessment of suitable patients is based on histological findings of endometrial samples along with MRI and/or transvaginal ultrasound images used for evaluation of myometrial invasion and extrauterine spreading [9,14].

Current guidelines suggest that EC patients younger than 50 years should be routinely evaluated for Lynch II syndrome [15]. However, there was just one such case in our study. To give more details of oncologic and obstetrical outcomes of Lynch syndrome patients a larger sample is needed in further research.

Hysteroscopy is the method of choice for diagnosis and treatment of endometrial cancer [8]. Direct tumor resection via hysteroscopy (three-step tumor resection) can remove the tumor quickly and effectively. Hysteroscopy is also used for evaluation (visualization and sampling) of endometrial response totherapy [14,16]. However, exploratory curettage of the uterine cavity is considered as more accurate than office-based hysteroscopy as it provides more tissue samples decreasing the chance of omitting the malignancy. Still, the number of curettages should be maximally reduced to minimize the endometrial damage (thinning and/or adhesions) which can negatively impact embryo implantation [9,14,15]. We assessed the endometrium both by hysteroscopy and curettage. We performed up to four interventions which were not found to affect conception.

Oral progestagen therapy is recommended by recent guidelines as the first line of treatment for EC in order to antagonize estrogen. It should be commenced immediately after surgery [3,5,17]. However, it can have different adverse effects that can lower the compliance of some patients [18,19]. GnRHa could be an alternative therapy that shows protective effects on ovarian reserve contributing to improved pregnancy rates, but it also has numerous side effects due to ovarian suppression. Consequently, GnRHa are currently suggested for obese patients and for duration up to six months [16,17,20]. Therefore, some protocols suggest using LNG-IUD which delivers a high concentration of progesterone directly to the endometrium while acting only locally, evading side effects. Studies have shownthat LNG-IUD therapy is associated with earlier disease regression [1,4]. LNG-IUD can be applied for prolonged periods, which is convenient as maintenance treatment. Thus, the combination of LNG-IUD with oral progestagens and/or GnRHa is currently the treatment of choice as it enhances treatment efficacy even more [5,19]. Our patients received LNG-IUD combined with GnRHa. Such treatment was initiated in our Clinic a decade ago as a somewhat experimental approach. As it was safe, effective, and comfortable, it is still the first-line treatment in our Clinic. We are currently complying with the new guidelines of LNG-IUD combined with oral progestagens [19].

Obesity as a part of the EC type 1-related metabolic syndrome is an important factor in endometrial malignant transformation [14]. Obesity decreases rates of complete remission. Moreover, diabetes mellitus is a frequent (30%) in obese EC patients. Therefore, weight loss is included into fertility-sparing protocols for all patients with BMI ≥ 25 kg/m^2^. Additional metformin therapy can improve outcomes for obese patients. Metformin upregulates progesterone receptors (making endometrium more sensitive to progesterone therapy), downregulates circulating insulin (insulin acts as growth factor for endometrial cancer cells), and inhibits proliferation and migration of endometrial cancer cells. Consequently patients on metformin therapy have shorter time to complete remission and lower recurrence rates [14,21,22]. Almost all of our patients with PCO syndrome and insulin resistance received metformin. However, in our sample PCO syndrome and diabetes mellitus were not common. Perhaps that was one of the reasons why there were no associations of endocrinological comorbidities with oncologic and obstetrical outcomes.

The literature shows that initial response of AH/EIN and EC to hormonal therapy is mostly very good (42% to 100% in different population). The response totherapy is better with combined treatment than if only a single agent is used. Rates of complete remission vary between studies and institutions, but are generally high, ranging from 53% to 75% [23]. Complete remission is, as expected, generally achieved more often for AH/EIN than EC (55% of EC and 82% of AH cases) and for progesterone-receptor-positive than -negative cases (60% to 72% for positive and 12% to 19% for negative cases) [24]. Patients treated with LNG-IUD and medroxyprogesterone acetate had complete remission in 87.5%, and the average time to complete remission was 9.8 ± 8.9 months (range 3 to 35 months) [8,10]. The authors report that if hysteroscopic resection of the tumor was primarily performed and then progestagen therapy was administered, a complete regression rate of 96.3% was achieved, with a median disease-free duration of 95 months (range 9 to 175 months). Consequently, some authors suggest that hormonal therapy should be administered for at least 9 to 12 months to enable long-term remission [14].

Still, recurrence rates even after successful completion of fertility-sparing treatment are mostly high and should not be underestimated [16,25]. The contemporary studies showed that recurrence rates vary between populations from 7.7% to 40.6%. The recurrence rates when only progestagens are used are higher (20% to 89%) than if combined therapy is applied (16%). In case of complete remission, long-term survival is relatively good, but if recurrence or progression are diagnosed the outcomes are generally adverse [9,26,27]. In our study complete remission rate was quite high (3/4 of patients). Still, recurrence after complete remission was registered in four patients. We also diagnosed three cases of progression from EIN to invasive EC. Out of all investigated characteristics of patients and malignancies, final oncologic outcomes were associated only with findings on regular controls.

Studies have found that the risk of a synchronous ovarian carcinoma in young EC patients ranges from 3% to 29%. Adnexal involvement decreases survival rates. However, the presence of two independent primary malignancies must be clearly distinguished from endometrial carcinoma spreading to the ovaries [28,29]. In our sample, there were two such patients who were both surgically treated.

After complete remission patient can be follow-up and attempt pregnancy. However, close surveillance of patients is necessary, and should include general and pelvic examination, endometrial sampling, tumor markers, and imaging of both the uterus and ovaries. Contrast-enhanced MRI is the most accurate method to detect myometrial invasion [15]. The achievement of pregnancy is the goal of any fertility-sparing therapy, as well as a marker of its success. Although the issue of pregnancy after fertility-sparing treatment of EC has been in focus for some time already, data regarding obstetrical outcome of such patients are still insufficient. Factors that can impact obstetrical outcomes are even less known. The literature shows that conception and live birth rates are promising, ranging from 20% if all treated patients are taken into consideration up to 93% if only patients who tried to conceive are analyzed. According to the literature out of patients who attempted pregnancy, maximal achieved pregnancy rate was 93.3% and live birth rate was 86.6%. However, studiesgenerally have shown that only around 50% of patients with complete EC remission attempt to conceive [10,11,15,30,31,32].

In our study, 29.9% of patients actually attempted to achieve pregnancy. The overall conception rate was low (around 12%), but if only women who tried to conceive were analyzed, the rate was almost 53%. The main factor that contributed to achieving pregnancy was obtaining complete remission in shorter time.

For pregnancy achievement, the majority of patients from the literature used ART [8,9,10,14]. Studies have also shown that shortening the interval between complete remission and assisted reproduction might be positively associated with a successful live birth [14,20]. ART was associated with higher pregnancy and live birth rates compared with spontaneous conception in young women with EC, most probably as they also had other risk factors of infertility. ART was found to be safe after complete remission of conservatively treated EC [14,23,33]. In our sample, no women who had ART had progressive or recurrent disease. Still, it should be pointed out that a significant number of pregnancies of our patients were spontaneously conceived.

Pregnancy can have a positive effect on the prognosis of EC. Recurrence-free survival is significantly longer and recurrence rates are lower (pregnancy 20.5% vs. no pregnancy 36.6%) if a patient achieves pregnancy after conservative EC treatment. A potential underlying mechanism is the fact that, during pregnancy, the uterus is under prolonged exposure to high concentrations of endogenous progesterone [33,34,35]. Our patients who achieved pregnancy also did not have progressive or recurrent diseases.

Advanced maternal age is generally a negative prognostic factor for both embryo implantation and live birth rates due to the physiologically decreased ovarian reserve and worse oocyte quality [9]. In our study, age was not a significant predictor of obstetrical outcome, although women with successful pregnancies were32 to 40 years of age.

It is important to point out that fertility-sparing treatment is still not considered as the standard care [33]. It is only a temporary measure, with the main goal of delaying definitive radical surgery to allow childbearing. Consequently, patients should be encouraged to get pregnant as early as possible when complete remission of disease is confirmed by histological examination. If the patient has no adequate partner, maintenance therapy with LNG-IUD is suggested. A definite surgery is strongly indicated after childbearing to avoid tumor recurrence [32,33,34,35,36].

The main limitation of this study was the relatively small number of patients. As the patients were investigated for a decade, some were lost in the follow-ups, as expected. However, fertility-sparing treatment is performed according to strict indications in specific patients, so it is difficult to obtain a large sample from a single institution. Our tertiary university clinic is the referral center for AH, EIN, and EC conservative treatment in Serbia, as well as the region. Our sample is therefore comparable in size with samples from other single-center studies. For endometrial cancer, long-term follow-up is essential to enable comprehensive data collection for adequate evaluation of patient outcomes [10,36]. Still, in this study, we focused on short- to medium-term outcomes, as we found them the most important due to the fact that short disease-free survival and quick recurrence of the disease could potentially prevent future fertility. Moreover, women who finished reproduction had treatment radicalization, and consequently their outcomes have changed and are no more associated with conservative but with radical treatment. Due to the small sample, we registered few cases of achieved pregnancy and even fewer deliveries. Still, in the past decade, conservative treatment of endometrial cancers was a very rare and basically experimental therapeutic approach, proposed to just a small number of patients according to strict rules and indications. Consequently, we could not expect to have numerous pregnancies of patients conservatively treated for EC. In the future, we plan to present data from longer follow-ups to maintain regularity of reporting data and novel findings, as well as to confirm obtained results in multicenter research.

Another limitation is the fact that not all of the patients received absolutely the same treatment. The treatment including LNG-IUD, GnRHa, metformin, and lifestyle changes can cause a bias in evaluation of the treatment method. Nevertheless, we administered treatments according to guidelines and recommendations, and consequently metformin with diet and physical activity was suggested only to patients who had indications for such therapy. In addition, this study is limited by the lack of acontrol group, as it would be very hard to form a control group. If acontrol group incorporated women treated due to AH/EIN and EC by standard radical surgery oncologic, outcomes would be superior, but such treatment would prevent future fertility. Consequently, obstetrical outcomes could not be compared between conservatively and radically treated patients. On the other hand, there are no alternative fertility-sparing treatments for AH/EIN and EC that could be compared with treatment administered to our patients.

Finally, the absence of the longer follow-up of newborns can raise questions of their long-term outcomes. However, as all newborns were healthy at the time of delivery, we do not expect any serious illnesses that might be caused by the maternal condition or its treatment. Further studies should be conducted to assess the condition of children born to mothers who were conservatively treated due to AH/EIN and EC.

## 5. Conclusions

Outcomes of patients treated according to current protocol for AH, EIN and early EC fertility-spearing therapy can be quite good in both terms of oncologic (high remission and low recurrence rates) and obstetric (achieved pregnancy and delivery of a healthy child) results. Prolonged disease remission was associated with absence of myometrial invasion and good histological finding of the control endometrial sampling. Successful pregnancy and a term delivery were associated with not having infertility issues prior to malignancy and achieving remission in a shorter time. A multidisciplinary approach is necessary to enable optimization and individualization of care for young women with AH, EIN, and/or EC.

## Figures and Tables

**Table 1:** General and medical history data of investigated women.

Parameters		Frequency	Percent	χ^2^	*p*
Cancer in family	no	60	69.0	12.517	0.001
	yes	27	31.0		
Body mass index	no obesity (<25 kg/m^2^)	48	55.2	0.931	0.335
	obesity (≥25 kg/m^2^)	39	44.8		
Menstrual cycle	regular	69	79.3	29.897	0.001
	not regular	18	20.7		
Comorbidity	no	57	65.5	8.379	0.004
	yes	30	34.5		
Other gynecological illnesses	no	74	85.1	167.391	0.001
	myoma	5	5.7		
	adenomyosis	4	4.6		
	ovarian tumors	4	4.6		
Papanicolaou test	II group	86	98.9	83.046	0.001
	III group	1	1.1		
Brest examination	no pathology	80	92.0	61.253	0.001
	dysplasia	7	8.0		
Polycystic ovarian syndrome	no	80	92.0	61.253	0.001
	yes	7	8.0		
Insulin resistance	no	76	87.4	30.265	0.001
	yes	11	12.6		
Infertility	no	44	50.6	0.011	0.915
	yes	43	49.4		

**Table 2:** Data regarding malignancy and treatment.

Parameters		Frequency	Percent	χ^2^	*p*
Diagnosis	AH in polyp	8	9.2	50.989	0.001
	AH	16	18.4		
	EIN in polyp	3	3.5		
	EIN	21	24.1		
	EC G1	33	37.9		
	EC G2	6	6.9		
Tumor markers	referral range	81	93.1	64.655	0.001
	increased	6	6.9		
Initial intervention	curettage	34	39.1	4.149	0.042
	hysteroscopy	53	60.9		
Control 6 months histopathology	complete remission	61	70.1	0.333	0.564
	persisting/progressing	26	29.9		
Further maintenance therapy up to 12 months	no	25	28.7	23.172	0.001
	IUD and GNRHa	49	56.3		
	+Metformin	12	13.8		
	+resection	7	8.0		
	radicalization	13	14.9		
Therapy response after 12 months	complete remission	65	74.7	21.253	0.001
	persisting/progressing	22	25.3		
Myometrial invasion signs on control MRI	no	76	87.4	48.563	0.001
	yes	11	12.6		
Final radicalization	no	60	81.1	28.595	0.001
	yes	14	18.9		

Legend: AH—atypical hyperplasia; EIN—endometrial intraepithelial neoplasm; EC—endometrial carcinoma; IUD—levonorgestrel releasing intrauterine device; GnRHa—gonadotropin-releasing hormone analogs; MRI—magnetic resonance imaging.

**Table 3:** Characteristics of women who had persistent or progressive AH, EIN, and/or EC.

Patient	Age	BMI	Infertility	Diagnosis	Condition After 12 Months	Follow-Up Therapy
1	38	24.70	yes	EC	persistent	IUD/GnRHa
2	38	23.40	yes	EC	persistent	IUD/GnRHa
3	27	24.09	no	polyp AH	persistent	IUD/GnRHa
4	41	27.90	yes	EC	persistent	IUD/GnRHa
5	26	24.20	no	EIN	persistent	IUD/GnRHa
6	37	24.50	yes	EC	persistent	IUD/GnRHa
7	28	25.80	yes	EIN	recurrent	IUD/GnRHa
8	41	21.90	yes	EIN	persistent	IUD/GnRHa
9	22	27.41	yes	AH	persistent	IUD/GnRHa
10	38	21.30	yes	AH	persistent	IUD/GnRHa
11	29	27.40	yes	EC	persistent	IUD/GnRHa
12	26	25.50	no	AH	recurrent	no
13	41	20.55	yes	EIN	progress	surgery
14	36	27.00	no	EC	recurrent	IUD/GnRHa
15	33	22.00	yes	EC	persistent	IUD/GnRHa
16	34	21.90	no	EIN	persistent	IUD/GnRHa
17	42	28.00	no	AH	persistent	IUD/GnRHa
18	37	24.00	no	EIN	progress	surgery
19	34	29.00	no	EIN	recurrent	no
20	34	30.00	no	EIN	persistent	IUD/GnRHa
21	28	28.00	no	EC	persistent	IUD/GnRHa
22	33	29.64	yes	EIN	progress	surgery

Legend: AH—atypical hyperplasia; EIN—endometrial intraepithelial neoplasm; EC—endometrial carcinoma; IUD—levonorgestrel releasing intrauterine device; GnRHa—gonadotropin-releasing hormone analogs.

**Table 4:** Obstetrical outcome of conservatively treated AH/EIN and EC patients.

Parameters		Frequency	Percent	χ^2^	*p*
Pregnancy attempted	no	61	70.1	38.753	0.001
	yes	26	29.9		
Pregnancy attempted by	assisted reproduction	17	65.4	2.462	0.117
	spontaneous	9	34.6		
Pregnancy succeeded	no	17	65.4	2.462	0.117
	yes	9	34.6		
Pregnancy conceived by	assisted reproduction	4	44.4	3.894	0.418
	spontaneous	5	55.6		
Pregnancy outcome	miscarriage	2	22.2	2.778	0.096
	healthy child	7	77.8		
Delivery	vaginal	2	28.6	1.286	0.257
	cesarean section	5	71.4		

**Table 5:** Characteristics of women who achieved pregnancy after AH, EIN, and EC treatment.

Patient	Age	BMI	Infertility	Diagnosis	Control Months	Control Findings	Follow-Up Therapy	Therapy Response
1	34	19.80	no	AH	6	malig-free	IUD/GnRHa	good
2	39	27.20	yes	EC	3	persistent	IUD/GnRHa	good
3	34	21.00	yes	AH	3	malig-free	no therapy	good
4	32	20.00	no	EC	6	malig-free	IUD/GnRHa	good
5	38	27.10	yes	EC	6	persistent	IUD/GnRHa	good
6	34	31.67	yes	polyp EIN	3	malig-free	no therapy	good
7	40	21.30	no	AH	6	malig-free	IUD/GnRHa	good

Legend: AH—atypical hyperplasia; EIN—endometrial intraepithelial neoplasm; EC—endometrial carcinoma; IUD—levonorgestrel releasing intrauterine device; GnRHa—gonadotropin-releasing hormone analogs; malig—malignancy.

**Table 6:** Regression analysis predictors of therapy response in conservatively treated patients with AH/EIN and EC.

Parameters		Coefficient B	Coefficient Wald	*p*	Odds Ratio	Lower 95% Confidence Interval	Upper 95% Confidence Interval
History data *p* = 0.481	Age	−0.037	0.644	0.422	0.964	0.882	1.054
	Body mass index	0.026	0.210	0.647	1.027	0.918	1.148
	Menstrual cycle	0.741	1.308	0.253	2.098	0.589	7.469
	Deliveries	−0.843	0.739	0.390	0.430	0.063	2.943
	Abortions	0.801	0.242	0.623	2.226	0.092	5.089
	Comorbidity	−0.081	0.019	0.891	0.922	0.290	2.931
	Infertility	0.116	0.046	0.830	1.123	0.392	3.215
	Cancer in family	−0.028	0.002	0.962	0.972	0.301	3.135
	Constant	−1.373	0.374	0.541	0.253		
Cancer data *p* = 0.037	Diagnosis AH/EIN/EC	−0.123	0.255	0.614	0.884	0.548	1.427
	Other pathology	0.025	0.002	0.968	1.026	0.299	3.515
	Tumor markers	−0.580	0.196	0.658	0.560	0.043	7.282
	Invasion	−2.468	0.005	0.799	0.002	0.001	1.001
	Controls number	−0.099	0.034	0.855	0.906	0.315	2.603
	Controls time	0.107	0.925	0.336	1.113	0.895	1.383
	Findings at 6 months	1.350	3.959	**0.047**	3.857	1.020	6.581
	Maintenance therapy	0.542	0.407	0.524	1.719	0.325	9.093
	Constant	−2.854	5.915	0.015	0.058		

**Table 7:** Regression analysis predictors of pregnancy achievement in conservatively treated AH/EIN and EC patients.

Parameters		Coefficient B	Coefficient Wald	*p*	Odds Ratio	Lower 95% Confidence Interval	Upper 95% Confidence Interval
History data *p* = 0.744	Age	0.095	1.614	0.204	1.100	0.949	1.275
	Body mass index	0.002	0.017	0.982	1.002	0.846	1.186
	Menstrual cycle	−0.670	0.305	0.580	0.512	0.048	5.509
	Deliveries	0.924	0.489	0.484	2.520	0.189	33.582
	Abortions	−8.829	0.045	0.999	0.003	0.001	2.001
	Comorbidity	0.035	0.002	0.967	1.036	0.196	5.480
	Infertility	0.496	0.391	0.532	1.642	0.347	7.778
	Constant	−5.079	1.891	0.169	0.006		
Cancer data *p* = 0.021	Diagnosis AH/EIN/EC	−0.526	1.308	0.253	0.591	0.240	1.456
	Other pathology	0.453	0.739	0.164	0.831	0.263	1.343
	Tumor markers	0.675	0.102	0.649	1.036	0.263	2.441
	Controls number	2.781	1.941	0.164	16.133	0.323	68.973
	Controls time	−3.701	2.245	**0.034**	0.496	0.198	1.241
	Findings at 6 months	1.919	1.727	0.189	6.817	0.389	19.369
	Maintenance therapy	−1.702	1.065	0.302	0.182	0.007	4.621
	Constant	5.115	0.031	0.037	8.431	2.147	43.813

## Data Availability

Data are available upon request from the authors due to patient confidentiality.
